# Subjective feelings associated with expectations and rewards during risky decision-making in impulse control disorder

**DOI:** 10.1038/s41598-024-53076-2

**Published:** 2024-03-04

**Authors:** Brittany Liebenow, Angela Jiang, Emily K. DiMarco, L. Paul Sands, Mary Moya-Mendez, Adrian W. Laxton, Mustafa S. Siddiqui, Ihtsham ul Haq, Kenneth T. Kishida

**Affiliations:** 1 Neuroscience Graduate Program, Wake Forest School of Medicine, Winston-Salem, NC USA; 2 Department of Translational Neuroscience, Wake Forest School of Medicine, Winston-Salem, NC USA; 3 Duke School of Medicine, Durham, NC USA; 4 Department of Neurosurgery, Wake Forest School of Medicine, Medical Center Boulevard, Winston-Salem, NC 27157 USA; 5 Department of Neurology, Wake Forest School of Medicine, Medical Center Boulevard, Winston-Salem, NC 27157 USA; 6 Department of Neurology, University of Miami Miller School of Medicine, Miami, FL USA; 7 Present Address: Fralin Biomedical Research Institute, Virginia Tech, Roanoke, VA 24016 USA

**Keywords:** Striatum, Decision, Cognitive neuroscience, Computational neuroscience, Emotion, Reward, Neuroscience, Parkinson's disease, Neurology, Neurological disorders, Movement disorders, Parkinson's disease, Psychology, Human behaviour, Learning algorithms, Computational models, Predictive medicine

## Abstract

Impulse Control Disorder (ICD) in Parkinson’s disease is a behavioral addiction induced by dopaminergic therapies, but otherwise unclear etiology. The current study investigates the interaction of reward processing variables, dopaminergic therapy, and risky decision-making and subjective feelings in patients with versus without ICD. Patients with (n = 18) and without (n = 12) ICD performed a risky decision-making task both ‘on’ and ‘off’ standard-of-care dopaminergic therapies (the task was performed on 2 different days with the order of on and off visits randomized for each patient). During each trial of the task, participants choose between two options, a gamble or a certain reward, and reported how they felt about decision outcomes. Subjective feelings of ‘pleasure’ are differentially driven by expectations of possible outcomes in patients with, versus without ICD. While off medication, the influence of expectations about risky-decisions on subjective feelings is reduced in patients with ICD versus without ICD. While on medication, the influence of expected outcomes in patients with ICD versus without ICD becomes similar. Computational modeling of behavior supports the idea that latent decision-making factors drive subjective feelings in patients with Parkinson’s disease and that ICD status is associated with a change in the relationship between factors associated with risky behavior and subjective feelings about the experienced outcomes. Our results also suggest that dopaminergic medications modulate the impact expectations have on the participants' subjective reports. Altogether our results suggest that expectations about risky decisions may be decoupled from subjective feelings in patients with ICD, and that dopaminergic medications may reengage these circuits and increase emotional reactivity in patients with ICD.

## Introduction

Impulse control disorder (ICD) is a class of behavioral addictions that occur in Parkinson’s disease (PD) as a side effect of dopaminergic therapies^1–6^. ICD consists of the sudden onset of risky decisions, including excessive gambling, shopping, sexual activity, and eating^1–5^. These ICD symptoms encompass behaviors associated with dynamic emotional states resulting directly from dopaminergic modulation^1–6^. Notably, dopaminergic processes underlie both substance use disorders and behavioral addictions, a general finding that led to gambling disorder as the first behavioral addiction described in the DSM-5^6–11^. It is unclear whether differences in dopaminergic processes or related behavior can be detected in individuals susceptible to developing ICD prior to administering dopaminergic agonist therapy. The ability to do so may provide an approach to identify patients susceptible to ICD prior to the onset of devastating symptoms and more generally may also provide a model for investigating dopaminergic systems in humans predisposed to addiction disorders.

Dopaminergic processes have previously been implicated in the modulation of subjective feelings and emotional changes induced by stimuli and substances in both behavioral and substance-based addiction disorders^12–15^. Positron emission tomography imaging has shown that patients with gambling disorder have increased dopamine release associated with higher levels of excitement^12^ while increased D2-receptor availability has been shown to be associated with modulations of subjective feelings in alcohol use disorder^13^. Dopamine transporter polymorphisms have been associated with changes in subjective responsiveness to amphetamines^14^. In patients with ICD, increased subjective ‘wanting’ was observed when patients were on dopaminergic therapies^15^. These and related findings raise questions regarding how subjective feelings in the context of risky – dopamine-system engaging – behavior may differ in individuals at risk for developing ICD, but also in the potential utility predictive measures of subjective feelings may have in identifying neurobehavioral conditions that predispose patients to developing addiction and substance use disorders more generally.

Rutledge and colleagues developed a computational model that is predictive of subjective feelings (e.g., subjective well-being or happiness) from objective and parametrically probed choice behavior^16–18^. They employed a risky decision-making task that required participants to choose (on each trial) between a certain reward or a gamble. Participants also report how they feel, which allowed the development of a computational model that links reward processing variables to measures of subjective feelings. The task and model have been validated and its utility demonstrated for estimating factors associated with positive subjective-feelings in healthy populations, patients with major depressive disorder, and patients with borderline personality disorder^16–19^. To our knowledge, this approach has not been applied to investigate risky decision-making and associated subjective feelings in patients with Parkinson’s disease and ICD (nor other behavioral addictions or substance use disorders).

Here, we apply a computational psychiatric approach^20,21^ to investigate risky decision-making behavior and associated subjective feelings in PD patients with versus without ICD while on and off their dopaminergic medication. We test the overarching hypotheses that patients with ICD have altered subjective feelings associated with risky decision-making and that dopaminergic modulation induces changes in how objective information drives feelings associated with subjective well-being. We show that subjective feelings about the outcomes of risky decisions in patients with ICD are less influenced by expectations when off dopaminergic medications, but that dopaminergic medications increase the weight of their prior expectations on their eventual subjective report. In contrast, patients without ICD show relatively higher influence of expectations on their subjective feelings, and that the weight of expectations are diminished when on their prescribed dopaminergic medications. We also observe a trend toward patients with ICD being more reactive to reward prediction errors about the outcomes of their risky choices, a potential effect that will require further work to verify. Here, we present our empirical findings, computational approach, and discuss the implications of our findings in relation to differences in patients with ICD and the role dopamine levels may play in modulating behavior and associated subjective feelings in patients with addiction disorders.

## Results

### Risk-taking in patients with ICD

ICD is characterized by a sudden increase in risky decisions caused by dopaminergic therapies. To test the hypothesis that patients with ICD will take more risks even when off medications, we compared risk-taking behavior in patients with ICD, versus patients without ICD, in an on- versus off-medication state. 18 patients with ICD and 12 patients without ICD each completed 2 visits: one visit while on their dopaminergic medication and one visit off their dopaminergic medication with the order of on- and off-medication visits randomized for each patient. The age, gender, medication status of the first visit, and the dopaminergic medications prescribed were not significantly different between the ICD and non-ICD groups (Supplementary Table 1).

**Figure 1:**
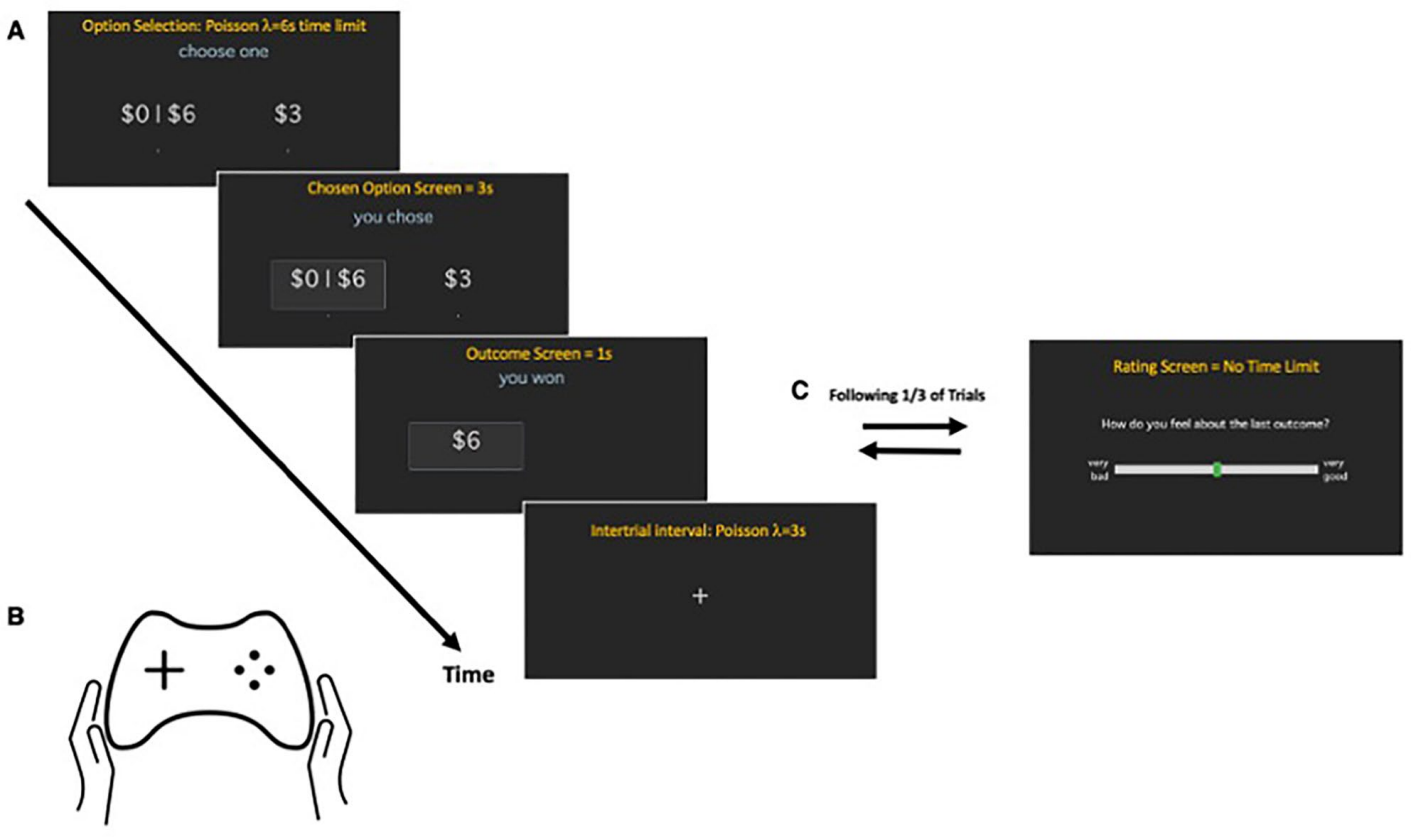
Timeline of events during the Sure Bet Or Gamble Task. The Sure Bet or Gamble (SBORG) Task is composed of independent trials (**A**) that participants interact with using a game controller (**B**) and computer screen. (**A**) On each trial, participants are asked to choose between a sure bet (a single number with 100% probability if selected) and a gamble (two numbers each with a 50–50% probability if selected). Consistent feedback about choice selection and outcome is given for each trial. Randomly, with 33% probability, participants are asked about their subjective feelings on a separate ratings screen (**C**). Note: the orange text is shown for descriptive purposes only; participants do not see the text shown in orange.

During each visit, patients completed a risky decision-making task consisting of multiple trials where they must choose between a certain reward option and a gamble option (Fig. 1). The decision to gamble occurred at the same rate in both groups (Supplemental Table 2) and was also not affected by medication state (off-medication, ICD = 47.00%, non-ICD = 46.04%, p-value = 0.9502; on-medication, ICD = 44.97%, non-ICD = 47.57%, p-value = 0.3048; ICD-on versus ICD-off, p-value = 0.7666; non-ICD-on versus non-ICD-off, p-value = 0.4023).

We fit a mixed-effects logistic regression model to participants’ decision to gamble with independent variables for the expected value of the gamble option, the value of the certain reward option, and the subjective feelings rating of the previous trial, and examined the differences in model coefficients between each group. Logistic regression models were fit separately for each participant and for both the on and off visits to account for individual variations in decision-making tendencies. We then examined the resulting model coefficients from each group. The model parameters from the logistic regression models revealed that, as expected, both groups’ decisions to gamble were positively influenced by the expected value of the gamble (${\beta }_{1}$: ICD off = 1.9517, ICD on = 2.2426, non-ICD off = 2.3155, non-ICD on = 2.1248) and negatively influenced by the value of the certain reward (${\beta }_{2}$: ICD off = −1.8869, ICD on = −2.0518, non-ICD off = −2.1320, non-ICD on = −1.9846) in both on and off medication states (Table 1). The prior trial’s subjective feelings rating did not significantly influence the decision to gamble in any group.

**Table 1:** Gamble choice model parameters. The “decision to gamble” on each trial was modeled as a dependent binary outcome with the independent variables being the “expected value of the gamble option”, “certain reward value”, and the participant’s actual or imputed “subjective feeling rating on the previous trial”, and a constant term “Baseline”. β_x_ – coefficients for each independent variable were fit in a logistic regression and reported.

OFF Medication	Non-ICD Off Medication				ICD Off Medication			
Model Parameter	Parameter Estimate	95% Confidence Interval	P-value	T-Statistic	Parameter Estimate	95% Confidence Interval	P-value	T-Statistic
Baseline (β_0_)	−0.2368	−1.241 to 0.767	0.614	−0.5191	0.0223	−0.772 to 0.817	0.953	0.0594
Gamble EV(β_1_)	2.3155	1.549 to 3.082	5.18E−05	6.7277	1.9517	1.360 to 2.544	3.05E−06	6.9895
Certain Reward EV(β_2_)	−2.1320	−2.932 to −1.332	0.000143	−5.9384	−1.8869	−2.506 to −1.267	7.91E−06	−6.4576
Subjective Feeling on (t−1) (β_3_)	0.0602	−0.445 to 0.566	0.796	0.2653	−0.2869	−0.836 to 0.262	0.284	−1.1071

ON Medication	Non-ICD On Medication				ICD On Medication			
Model Parameter	Parameter Estimate	95% Confidence Interval	P-value	T-Statistic	Parameter Estimate	95% Confidence Interval	P-value	T-Statistic
Baseline (β_0_)	−0.2583	−1.529 to 1.013	0.660	−0.4528	−0.1182	−1.199 to 0.962	0.820	−0.2309
Gamble EV(β_1_)	2.1248	1.644 to 2.605	1.82E−06	9.8556	2.2426	1.553 to 2.932	2.76E−06	6.8611
Certain Reward EV(β_2_)	−1.9846	−2.628 to −1.342	4.31E−05	−6.8774	−2.0518	−2.668 to −1.436	2.04E−06	−7.0252
Subjective Feeling on (t−1) (β_3_)	−0.1009	−0.305 to 0.103	0.296	−1.1016	−0.0936	−0.327 to 0.139	0.407	−0.8511

### Predictors of subjective feelings differentiate ICD-status in off-medication state

**Figure 2:**
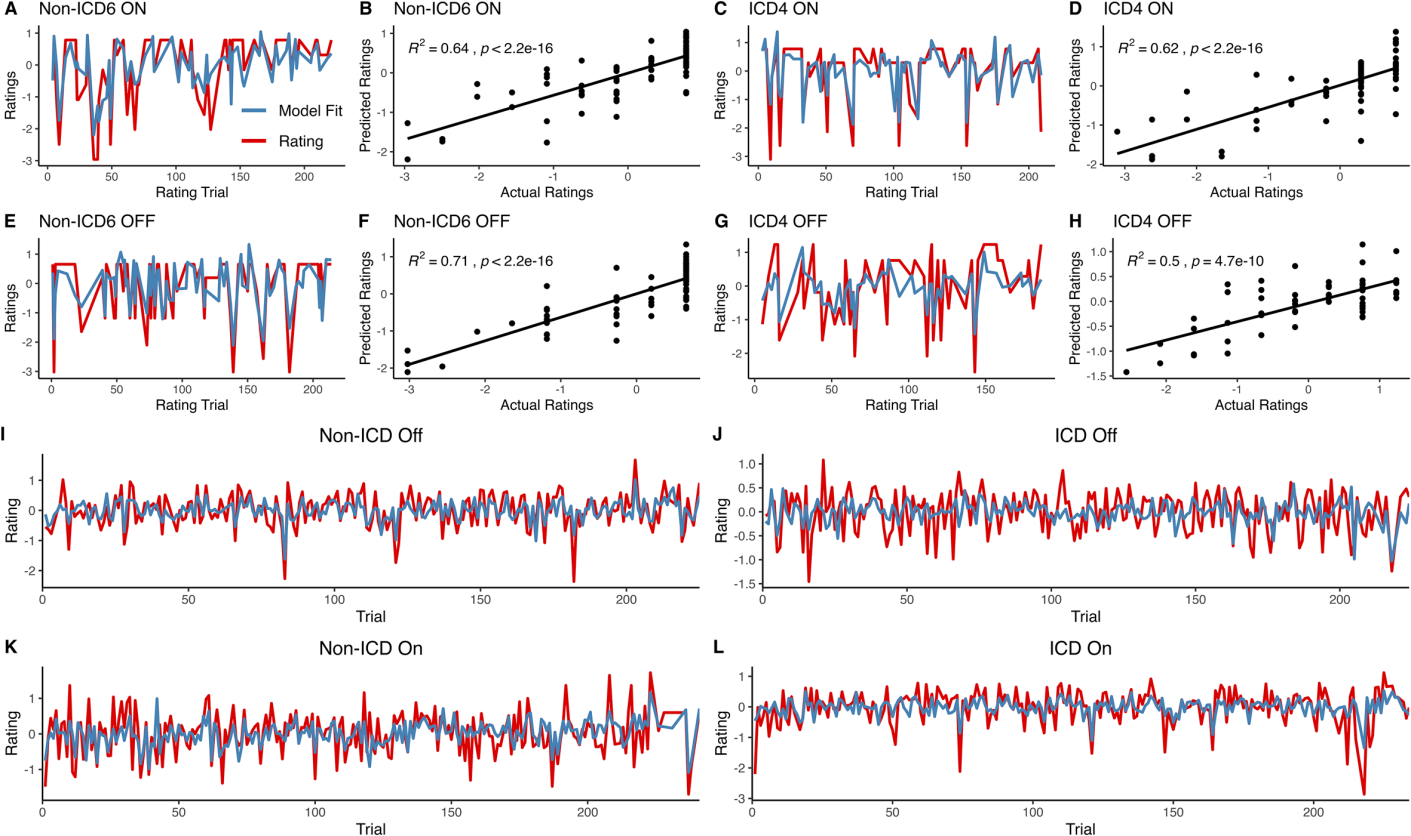
Happiness model performance: The happiness model was fit using hierarchical Bayesian analysis to estimate both individual-level and group-level parameters simultaneously. The model’s predicted ratings appeared to follow participants' actual ratings. (**A–H**) Selected non-ICD and ICD individual participants’ ratings and the model’s predicted ratings are plotted for their on-medication visit (**A**, **C**) and off-medication visit (**E**, **G**). The resulting correlation between the actual and predicted ratings are shown in (**B**, **D**, **F**, **H**). Mean ratings and model predicted ratings across subjects in each group: (**I**) Non-ICD Off: r^2^ = 0.3888, (**J**) ICD Off: r^2^ = 0.1759, (**K**) Non-ICD On: r^2^ = 0.3698, (**L**) ICD On: r^2^ = 0.2663.

We next tested the hypothesis that the influence of objective decision-making variables on participants’ subjective experience would be different in patients with a history of ICD versus those without (Table 2). During the task, participants were instructed to rate their subjective feeling of the outcome after a third of randomly selected trials. We fit Rutledge’s happiness model^16^ using a hierarchical Bayesian approach to behavioral data from each participants’ off-medication and on-medication visits. The happiness model parameters consist of an intercept term (w_0_) which serves as a baseline; a weight for the value of the certain reward option (w_1_) if the certain reward is chosen on a particular trial; a weight for the expected value of the gamble option (w_2_) if the gamble is chosen in a trial; a weight for the reward prediction error (w_3_), and a forgetting factor ($\gamma$) which modulates the extent to which events in past trials impact subjective feelings in the current trial. Parameters in the hierarchical model were simultaneously fit to each individual, as well as on a group level to the ICD and non-ICD groups, resulting in 2 sets of parameters: individual-level parameters which describe the behavior of each individual in the group during their off-medication visit, and group-level parameters which characterize the ICD and non-ICD groups as a whole when off their medication. Individual-level parameters were used to evaluate model fit by comparing the model’s predicted ratings to participants’ ratings. The model fit well to participant data (Fig. 2) with r^2^ = 0.3888 for the non-ICD group and r^2^ = 0.1761 for the ICD groups’ off-medication visit. To examine differences between groups, we compared the group-level parameter sets by evaluating the effect size (Cohen’s *d*), 95% highest density intervals (HDI), and credible values between each groups’ posterior distribution.

**Table 2:** Happiness model group-level parameter comparisons: ICD versus non-ICD. The 95% highest density interval (HDI) for each parameter’s posterior distribution is reported to summarize each distribution. Cohen’s d was used to measure the difference between posterior distributions in each group and provide an estimate the effect size between ICD and non-ICD groups. The 95% HDI and credible differences for the posterior distribution of differences between ICD-off versus non-ICD-off (3a) and ICD-on versus non-ICD-on (3b) are reported.

(a) OFF Medication		Baseline (w0)	Certain Reward (w1)	Gamble EV (w2)	RPE (w3)	Recent Experience Weight (γ)
ICD Off Medication	Parameter Estimate	−0.6243	0.1372	0.1058	0.3298	0.1754
	95% Highest Density Interval	−0.860	0.098	0.058	0.267	0.003
		to	to	to	to	to
		−0.407	0.178	0.157	0.392	0.322
Non-ICD Off Medication	Parameter Estimate	−1.1564	0.2418	0.2261	0.4602	0.1414
	95% Highest Density Interval	−1.379	0.196	0.176	0.331	0.038
		to	to	to	to	to
		−0.931	0.289	0.278	0.589	0.24
ICD Off : non-ICD Off	Difference in Means (Cohen’s d)	4.6206	−4.7254	−4.7147	−2.5440	0.4785
	95% Highest Density Interval	0.2081	−0.1637	−0.1897	−0.2743	−0.1535
		to	to	to	to	to
		0.8455	−0.0412	−0.0486	0.0115	0.2330
	% Credible differences greater & less than 0	0.05% < 0 < 99.95%	99.94% < 0 < 0.06%	99.97% < 0 < 0.03%	96.45% < 0 < 3.55%	37.66% < 0 < 62.34%

(b) ON Medication		Baseline (w0)	Certain Reward (w1)	Gamble EV (w2)	RPE (w3)	Recent Experience Weight (γ)
ICD On Medication	Parameter Estimate	−0.8422	0.1902	0.1657	0.3459	0.1434
	95% Highest Density Interval	−1.035	0.152	0.122	0.238	0.035
		to	to	to	to	to
		−0.642	0.226	0.209	0.461	0.245
Non-ICD On Medication	Parameter Estimate	−1.0175	0.2231	0.1914	0.4453	0.165
	95% Highest Density Interval	−1.253	0.182	0.130	0.323	0.068
		to	to	to	to	to
		−0.790	0.264	0.253	0.575	0.260
ICD On : non-ICD On	Difference in Means (Cohen’s d)	1.5883	−1.6343	−0.9442	−1.6512	−0.4204
	95% Highest Density Interval	−0.1323	−0.0873	−0.0994	−0.2665	−0.1668
		to	to	to	to	to
		0.4794	0.0246	0.0527	0.0708	0.1175
	% Credible differences greater & less than 0	13.27% < 0 < 86.73%	87.35% < 0 < 12.65%	74.81% < 0 < 25.19%	88.62% < 0 < 11.38%	61.53% < 0 < 38.47%

**Figure 3:**
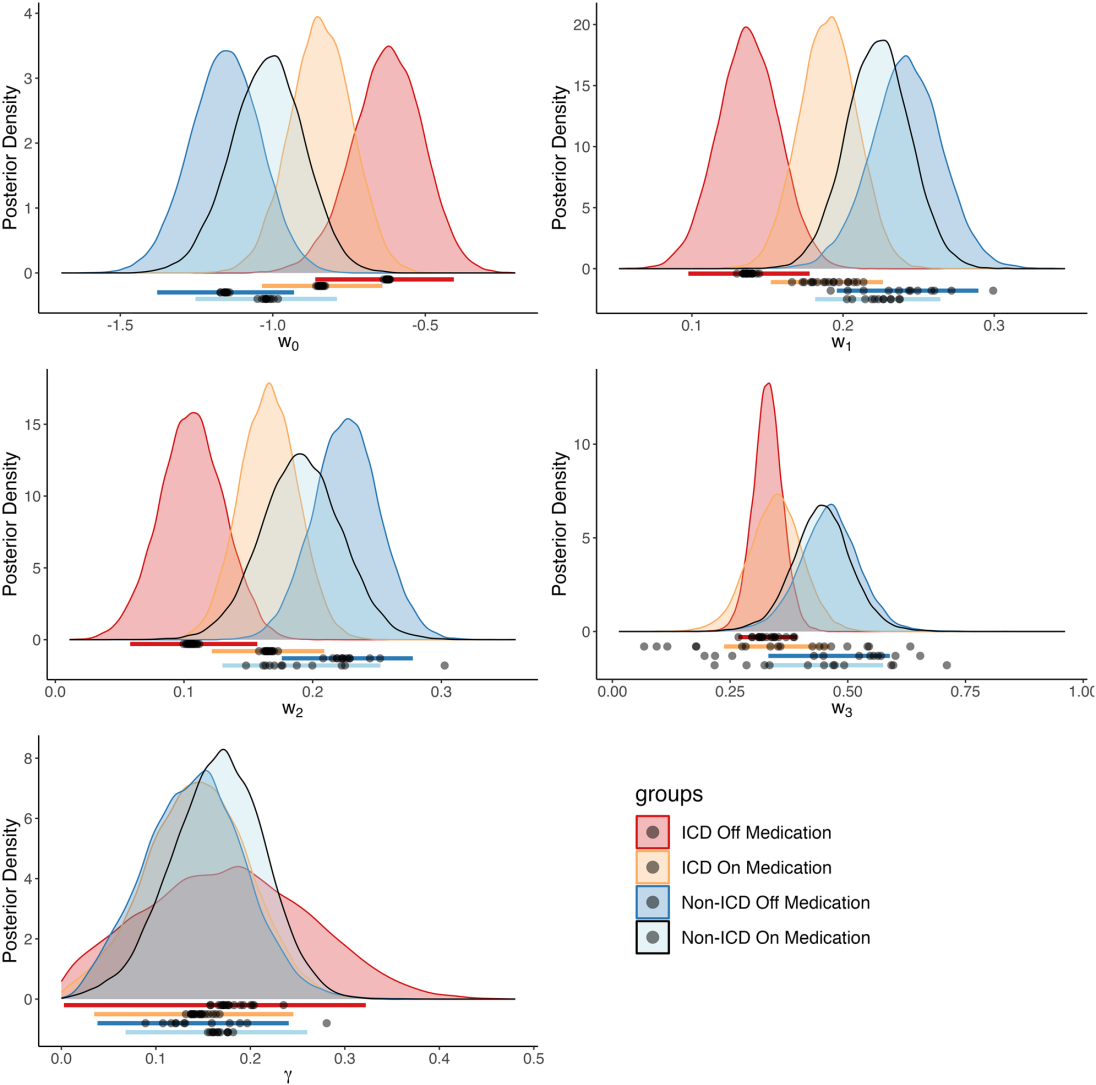
Posterior distributions over group-level happiness model parameters. Each subplot displays the distributions for a distinct parameter: the intercept (w_0_), the value of the certain reward when chosen (w_1_), the expected value of the gamble option when chosen (w_2_), the reward prediction error (w_3_), and the forgetting factor (γ). The colored lines below each distribution mark the 95% highest density intervals for each group, and are colored accordingly. Individual parameter means estimated by the model are marked by points below the posterior distributions.

**Figure 4:**
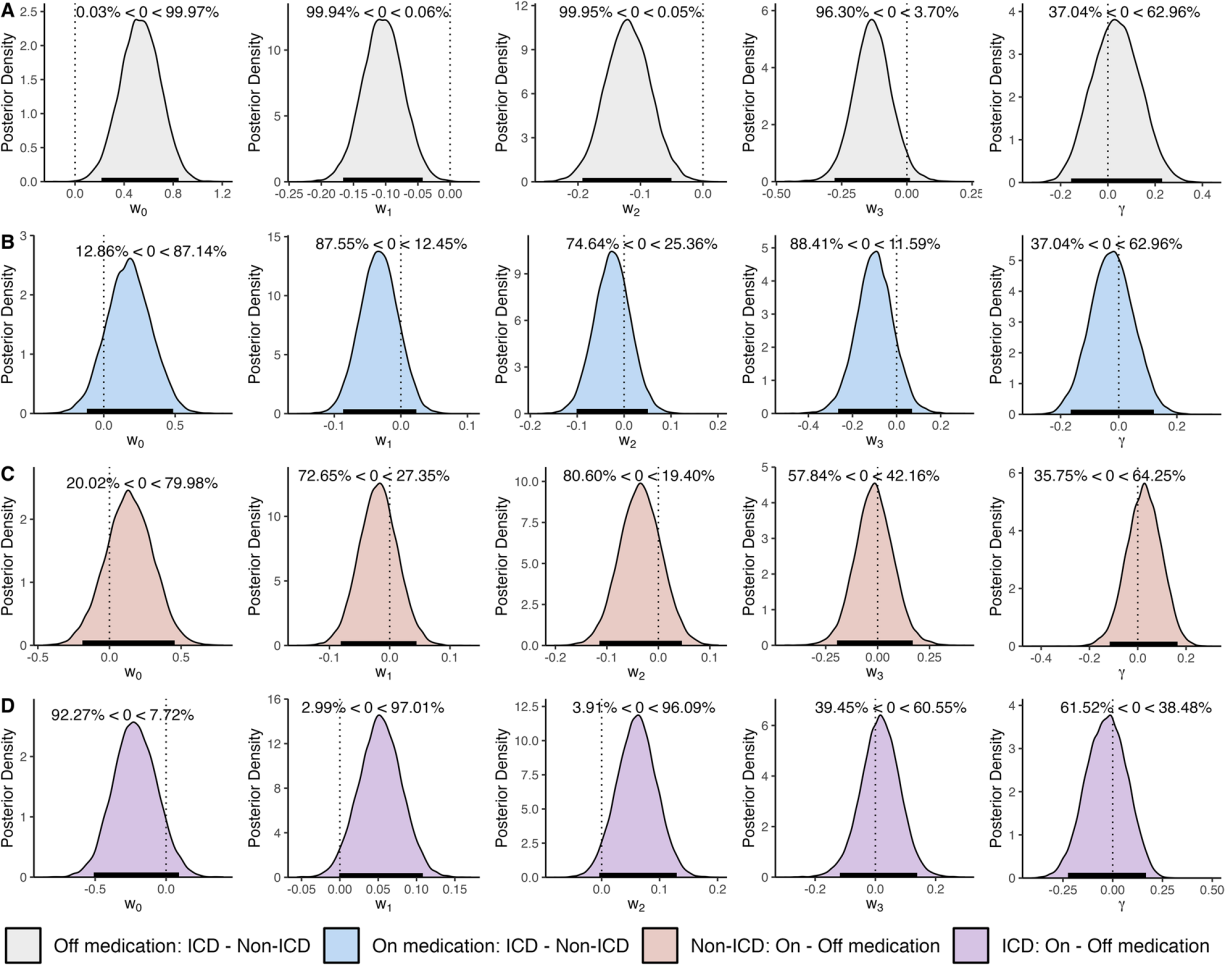
Difference of means. To compare how the ICD and non-ICD groups differ, we report the difference between the ICD and non-ICD groups’ posterior distributions when Off medication (Row A) and when On medication (Row B). For the effect of dopaminergic medication on each group, we report the difference of means between on and off medication posterior distributions for the non-ICD group (Row C) and ICD group (Row D). 95% highest density intervals are marked by a horizontal line at the base of each distribution.

Subjective feeling (i.e., ‘Happiness’) model parameters for patients in the off-medication state were significantly different across ICD and non-ICD groups as observed in the posterior distributions for each parameter (Fig. 3, Table 2a). The baseline weight was larger in ICD compared to non-ICD, and the influence of the value of the certain reward and the expected value of the gamble were smaller in the ICD group. 95% HDIs, which indicate which points of a distribution are most likely to contain the true value, were non-overlapping for the baseline weight (w_0_, ICD HDI: [−0.860, −0.407], non-ICD HDI: [−1.379, −0.931]), the certain reward term weight (w_1_, ICD HDI: [0.098, 0.178], non-ICD HDI: [0.196, 0.289]), and the weight for the expected value of chosen gambles (w_2_, ICD HDI: [0.058, 0.157], non-ICD HDI: [0.176, 0.278]). To gain a better understanding of these differences, we report the posterior distribution of differences between the ICD and non-ICD group when off medication which show a clear separation between how the two groups weigh the certain reward, the expected value of chosen gambles, and the baseline (Fig. 4a). The 95% HDI of the difference of means fell below 0 for the w_1_, and w_2_ terms (w_1_: [−0.1661, −0.0426], w_2_: [−0.1929, −0.0505]). The HDI, along with the probability of credible differences less than zero (Off medication: P(ICD w_1_ – non-ICD w_1_ < 0) = 99.94%, P(ICD w_2_ – non-ICD w_2_ < 0) = 99.95%), indicate these weights are smaller in the ICD group compared to the non-ICD group. The baseline weight (w_0_) was credibly larger in the ICD group than the non-ICD group (95% HDI: [0.2158, 0.8443], P(ICD w_0_ – non-ICD w_0_ > 0) = 99.97%). Group-level parameters also showed large Cohen’s *d* effect sizes (magnitude > 0.8) between the ICD and non-ICD groups’ baseline (*d* = 4.6206), certain reward (*d* = −4.7254), and gamble expected value (*d* = −4.7147) weights (Table 2a).

In both the on- and off-medication state, the weight of the reward prediction error term on subjective feelings trended towards significant differences between the ICD versus non-ICD groups irrespective of the medication status. Notably, the difference approached significance, but did not meet strict criteria. In both the on- and off- state, patients with ICD may show less influence of the reward prediction error feedback signal compared to patients without ICD. Specifically, when patients were on medication, the 95% HDI values for the reward prediction error term were [0.238, 0.461] for the ICD group and [0.323, 0.575] for the non-ICD group. Similarly, in the off-medication state, the HDI values were [0.267, 0.392] for the ICD group and [0.331, 0.589] for the non-ICD group (Table 2). Further analysis of the posterior distribution of differences indicates an approaching significance between the ICD and non-ICD groups for the on-medication state (95% HDI: [−0.277, 0.012], P(ICD w_3_ – non-ICD w_3_ < 0) = 96.30%, Fig. 4A) and off-medication state (95% HDI: [−0.265, 0.070], P(ICD w_3_ – non-ICD w_3_ < 0) = 88.41%, Fig. 4B). While these values do not meet the conventional thresholds for significance (i.e., cutoff at 97.5%), their proximity to this threshold suggests a strong trend, which may be revealed in future studies with a sample size sufficient to detect what may be a smaller effect than the current study could differentiate. Though, Cohen’s *d* effect sizes indicate a relatively large magnitude (Off medication: d = −2.544, On medication: d = −1.6512).

### Predictors of subjective feelings in ICD and non-ICD groups converge in on-medication state

The addition of dopaminergic medication changed the influence of gamble decision-making variables in both ICD and non-ICD groups. Notably, the impact of these changes was that the influence of expected outcomes for both groups became more similar when on medication. Patients with ICD showed an increase while patients without ICD showed a decrease (Fig. 3).

We fit the subjective feeling model to the ICD and non-ICD groups’ on-medication visit behavior (Non-ICD r^2^ = 0.3699, ICD r^2^ = 0.2663, Fig. 3) and compared the parameter estimates between the two groups (Fig. 4b). No differences between groups were observed in the weights for the baseline (ICD – non-ICD: 95% HDI = [−0.1323, 0.4794], Cohen’s *d* = 1.5883, P(ICD w_0_ – non-ICD w_0_ > 0) = 86.73% Table 2b), the certain reward (ICD – non-ICD: 95% HDI = [−0.0873, 0.0246], Cohen’s *d* = −1.6343, P(ICD w_1_ – non-ICD w_1_ < 0) = 87.35% Table 2b), the expected value of the gamble (ICD – non-ICD: 95% HDI = [−0.0994, 0.0527], Cohen’s *d* = −0.9442, P(ICD w_2_ – non-ICD w_2_ < 0) = 74.81%, Table 2b), the reward prediction error (ICD – non-ICD: 95% HDI = [−0.2665, 0.0708], Cohen’s *d* = −1.6512, P(ICD w_3_ – non-ICD w_3_ < 0) = 88.62%, Table 2b), and the influence of the recent experience on subjective feelings (ICD – non-ICD: 95% HDI = [−0.1668, 0.1175], Cohen’s *d* = −0.4204, P(ICD $\gamma$ – non-ICD $\gamma$ < 0) = 61.53%,Table 2b).

### Dopaminergic medications do not differentially influence predictors of subjective experience in ICD and non-ICD groups

The expected value of chosen gambles, collection of certain rewards, and reward prediction errors following chosen gambles are hypothesized to engage or be affected by the dopaminergic system; thus, we hypothesized that the impact of these variables on subjective experience would be modulated by dopaminergic medications used to treat PD symptoms (Table 2b).

We examined how behavior changes between on and off medication visits. In patients without ICD, the weights for the baseline, influence of the certain reward, and the expected value of the gamble were not credibly different in the on-medication state compared to the off-medication state (Fig. 3, Fig. 4c, Table 3a). The influence of the certain reward (On – Off: 95% HDI = [−0.0798, 0.0447], Cohen’s *d* = −0.8307, P(On w_1_ – Off w_1_ < 0) = 72.61% Table 3a) and expected value of the gamble shifted lower when on medication compared to off medication (On – Off: 95% HDI = [−0.1139, 0.0453], Cohen’s *d* = −1.2073, P(On w_1_ – Off w_1_ < 0) = 80.68% Table 3a), and the baseline weight was greater when patients were on medication (On – Off: 95% HDI = [−0.1890, 0.4564], Cohen’s *d* = 1.1891, P(On w_0_ – Off w_0_ > 0) = 79.91% Table 3a). In patients with ICD (Fig. 4d), the direction of the changes was reversed, though the changes were also not credibly different based on a 95% HDI. When on medication, the influence of the certain reward (On – Off: 95% HDI = [−0.0011, 0.1085], Cohen’s *d* = 2.6847, P(On w_1_ – Off w_1_ > 0) = 97.13% Table 3b) and the expected value of the gamble (On – Off: 95% HDI = [−0.0055, 0.1278], Cohen’s *d* = 2.5095, P(On w_2_ – Off w_2_ > 0) = 96.24% Table 3b) increased in magnitude; while the baseline decreased (On – Off: 95% HDI = [−0.5152, 0.0862], Cohen’s *d* = −2.0075, P(On w_0_ – Off w_0_ < 0) = 92.07%, Table 3b). The influence of the reward prediction error and influence of recent experience did not change greatly in either patient group (Table 3a and Table 3b).

**Table 3:** Happiness model group-level parameter comparisons: On versus Off medication. Participants’ ‘happiness’ with their decision outcomes were modeled as the dependent variables using Eq. 1. Parameter weight estimates for each group (ICD and non-ICD) and medication state (on and off) were determined using hierarchichal bayesian modeling (Table 2). Cohen’s *d* effect sizes and highest density intervals (95% HDI) for comparisons across non-ICD-on versus non-ICD-off (3a) or ICD-on versus ICD-off (3b) with corresponding credible differences are reported.

(a) non-ICD On : non-ICD Off	Cohen’s *d*	95% Highest Density Interval	% Credible Differences
Baseline (w0)	1.1790	−0.1887 to 0.4554	20.02% < 0 < 79.98%
Certain Reward (w1)	−0.8350	−0.0811 to 0.0445	72.65% < 0 < 27.35%
Gamble EV (w2)	−1.2096	−0.1144 to 0.0455	80.60% < 0 < 19.40%
RPE (w3)	−0.2568	−0.1955 to 0.1688	57.84% < 0 < 42.16%
Recent Experience Weight (γ)	0.5022	−0.1160 to 0.1647	35.75% < 0 < 64.25%

(b) ICD On : ICD Off	Cohen’s *d*	95% Highest Density Interval	% Credible Differences
Baseline (w0)	−2.0227	−0.5092 to 0.0905	92.27% < 0 < 7.72%
Certain Reward (w1)	2.6644	−0.0008 to 0.1082	2.99% < 0 < 97.01%
Gamble EV (w2)	2.5143	−0.0040 to 0.1296	3.91% < 0 < 96.09%
RPE (w3)	0.3491	−0.1178 to 0.1387	39.45% < 0 < 60.55%
Recent Experience Weight (γ)	−0.4497	−0.2247 to 0.1672	61.52% < 0 < 38.48%

## Discussion

ICD is a behavioral addiction disorder caused by dopaminergic action^3,22^, and dopaminergic systems are engaged by reward expectation, reward feedback, and associated subjective feelings^18,23–25^. Thus, we sought to understand the impact dopaminergic medications and ICD status may have on decisions to take risks and subjective feelings associated with these actions. In our experimental setting, the rate of risky decisions was not different across ICD and non-ICD groups nor affected by the medication state (Supplemental Table 2). However, the influence of expectations and reward processing variables on subjective feelings significantly differed in the ICD group compared to the non-ICD group when off medication. When both groups were on medication, this influence no longer differed significantly. Notably, the influence of feedback (i.e., the reward prediction error) appears diminished in patients with ICD, regardless of medication state; however, the potential differences in this parameter were borderline and did not meet a strict 95% HDI statistical threshold. Altogether, our results suggest that patients with ICD may be in a predisposed state where risky choices and dopaminergic medications express a differential influence on emotional states compared to patients without ICD. Our results are consistent with the hypothesis that the impact of ICD induced by dopamine receptor agonistst may reveal enhanced emotional reactivity associated with increased risky behavior driven by expectations and less influenced by consequences. Further work is needed to determine whether there is a statistically significant difference in the influence of reward prediction errors across patients with versus without ICD. And, future work should explore whether a computational psychiatric approach to estimating patients’ emotional reactivity to gambling behaviors could be used as a prognostic biomarker for patients at increased risk of developing ICD or perhaps other addiction disorders.

We did not find clear model evidence to support a difference in parameters between the on and off medication states within the ICD or non-ICD group. However in the ICD group, we observed that the probability that the parameters are different at w_0_ = 92.27% , w_1_ = 97.01%, w_2_ = 96.09%, which suggest a strong likelihood of an actual difference between the two groups. This, and the fact that the difference in parameters in the on- vs off-medication states had large Cohen’s *d* effect sizes implies that replicating the study with a larger sample size is warranted to gain confidence in the signifcance of these results.

The observation that patients with ICD take an equal number of risks as patients without ICD (independent of medication state) is consistent with our experimental design and prior work^26^, though seemingly contradictory to expectation given the ICD phenotype^3,22,26^. On any given trial there is a 50% chance that the gamble option is rationally the better option. That participants chose to gamble slightly less than 50% of the time is consistent with the general phenomena of humans being risk averse^27,28^. We conducted further analysis on trials where the gamble option was not clearly optimal, where the value of the certain reward option exceeded the expected value of the gamble option (Supplementary Table 3). However, we did not observe any significant differences in the rate at which each group chose to gamble. Notably, participants experienced a number of conditions where either the gamble or the sure bet are clearly the best choice (e.g., sure bet option is greater than either gamble outcome or gamble outcomes are both better than the sure bet option). These were included to allow a control for participants’ behavior – to determine whether they continue to choose rational outcomes throughout the task. These ‘control trials’ may have altered the context of the more conflicting gamble versus sure bet trials or may have given participants more experience with the gamble options and thus a better experience-based understanding of the true nature of the 50/50 gamble. Either of these explanations may explain why participants do not express a gambling behavior effect in our study. However, our design and the resulting lack of gambling behavior effects enable us to investigate and report an effect on subjective feelings that may otherwise had been confounded with variable rates of gambling behavior. Our computational psychiatric approach^20,21^ and utilization of Rutledge and colleagues’ computational model of happiness^16–18^ allowed for a more precise investigation into factors that influence dynamic changes in subjective feelings associated with risky choices in the context of a behavioral addiction disorder.

Dopaminergic medications are expected to modulate how reward-related variables are processed by dopaminergic systems including the role those systems may play in the generation of emotion. Indeed, when patients with ICD are on their prescribed dopaminergic medications, the influence of the expected value of the gamble on their feelings increases, as does the influence of the value of the certain reward. This result is consistent with dopaminergic medications increasing emotional reactivity in the ICD group, as the increase in the weight values indicate patients become more sensitive to the expected values. In patients without ICD, these parameters saw little change between medication states, indicating dopaminergic medication did not influence how much certain reward and expected value of the gamble influenced their feelings. Patients without ICD may be in a state that diminishes or prevents the effects of the medication observed in the ICD group. The evaluation of model fit revealed that the model exhibited comparatively poorer fit in the ICD off medication group (r^2^ = 0.1761) when compared to the other groups (ICD On = 0.2663, Non-ICD Off = 0.3890, Non-ICD On = 0.3699). This suggests that when off medication, the ICD cohort may experience subjective feelings that are less predictable by these factors. Alternatively, this cohort may have decreased emotional awareness and were less accurately reporting their true feelings during the task when off medication. However, additional investigation may be of interest to explore this specific possibility in greater depth as the task and model were not specifically designed to disentangle this possibility. The increased volatility observed in subjective ratings when off medication as well as the increased valuation of chosen options when on medication could potentially signify underlying neurobiological differences that may contribute to the development of ICD and may be used to detect PD patients at risk of developing ICD prior to the initiation of medication regimens.

In the present experimental context computational modeling revealed subtle differences that would otherwise be difficult to detect. This suggests potential for future work to develop a behavioral screening approach^15^. The observed differences in parameters between the ICD and non-ICD groups in the off-medication state, along with their convergence when on medication, offer promising indications that a task of this nature holds potential for distinguishing between individuals susceptible to developing ICD and those less likely to do so. The participants in this study have already experienced a standard of care approach to determine their medication strategy. In this process these patients have in the past or were currently positive for ICD. Thus, we cannot determine whether our results for patient in the off-medication state are reflective of their predisposition or if these results reflect the consequences of having already experienced an ICD-inducing medication. Further study is needed to examine whether the use of model parameters can be used to predict if a patient is prone to developing ICD following the prescription of specific dopaminergic medications. The task we use is short, only 30 min, and can likely be significantly shortened. Development of such a screening tool would be a valuable addition to other more complex multi-session interactions or neuroimaging based approaches^29,30^. More work is needed before such a tool could be implemented, but our results suggest potential utility in using objective measures of moment-to-moment changes in subjective feelings as expressed through task behavior.

There is a yet unclear and complex relationship between reward processing variables, associated subjective feelings, the dopaminergic system, and disorders like PD and ICD. Through computational modeling of risky decisions, we identify quantitative changes in the influences of reward processing on subjective feelings and how these systems may be altered by dopaminergic medications and ICD. Our results are consistent with risk taking in patients with ICD being a form of significantly altered behavior with associated changes in subjective experience that is affected by stimuli and interventions that engage the dopaminergic system. Our results also suggest that a computational psychiatric approach may be able to identify patients at risk for developing ICD or perhaps other addiction disorders, but more work is needed before this conclusion can be reached. The present study was conducted at a single center; it is possible this introduces bias into our study that we cannot measure or detect. Similarly, a single-center study places a limit on sample size; though, our sample is comparable to previous studies^26^. Prior to data collection, a power analysis was conducted on preliminary data from a prior study involving the same patient population (i.e., patient with PD with and without ICD) performing a risky decision-making task to determine an estimate of an appropriate sample size. A sample size of 30 was determined to be sufficient to detect large effects. However, the present data are novel in the combination of patient population, experimental manipulations, and specific behavioral tasks and analyses. A larger sample size in future work may yield more insight into the processes involved in relating behavior to subjective feelings and individual differences to be observed in individuals with an addiction disorder. Nonetheless, our results highlight the impact computational precision can have in aiding our understanding of complex, dynamic behaviors associated with risk taking, reward processing, and subjective experience in psychiatric conditions like ICD and provide evidence more generally that latent variables that characterize subtle dynamic changes in experience may be used to better understand the mechanisms underlying human experience and behavior.

## Methods

The Institutional Review Board at Wake Forest University Health Sciences approved all procedures described (protocol #: IRB00051643). All research was performed in accordance with relevant guidelines including those outlined in the Declaration of Helsinki. Informed consent was obtained from all participants.

### Participants

Patients with PD were recruited from the Movement Disorders Clinic at Atrium Health Wake Forest Baptist. The Questionnaire for Impulsive-Compulsive Disorders in Parkinson’s Disease-Rating Scale (QUIP-RS) was used to determine ICD status^4^. A cutoff of 10 or higher on the ICD categories of the QUIP-RS (gambling, sex, buying, and eating) was used to sort patients into the ICD group (N = 18), and PD patients scoring less than 10 were sorted into the non-ICD group (N = 12)^4,5^. Patients in the ICD and non-ICD groups were matched for gender and age and standard of care medications recorded (Supplemental Table 1).

### Procedures

All participants were scheduled for two research study visits. On both visits, the participants performed the risky decision-making task (Fig. 1). Participants were instructed to come to the study visit in either an on- or off-medication state – the order randomized across participants. For the on-medication state, participants were instructed to take their medications as usual. For the off-medication state, participants were instructed to withhold from taking medications for at least 8 h prior to their scheduled session. Participants were told to withhold their medications with the prior night’s dose being the last they should have taken and arrived in the clinic setting off medications in the morning. Only verbal confirmation was used to confirm adherence to the requested medication state. However, the severity of patients’ motor function in the off-medication state (particularly compared to their on-medication state) was visually evident. During their on-medication visit, participants completed significantly more trials, which is expected due to the increased difficulty of initiating movement required to press the controller button when off-medication. This suggests that participants were compliant with following instructions to withhold their dopaminergic medication prior to their off-medication visit (Supplementary Table 4).

#### Risky decision-making task (Fig. 1)

Participants complete a risky decision-making task delivered through a computer-controlled interface (Fig. 1). This task is based on Rutledge and colleagues’ prior work^16–18^: On each trial, participants are presented with two possible gain options: a certain reward or a gamble. Certain reward represents a value equal to a shown dollar amount ranging $1 to $6, and gamble represents two independent values ranging $0 to $6 each with a 0.5 probability of payout. The side for each option is randomized for each trial. Decisions must be made ‘as quickly as possible’ with a time limit set according to random draws from a Poisson distribution with $$\lambda =6s$$. If participants failed to respond within the time limit, they receive a screen with the message “too late” (this rarely happened, 2.96% out of an average of 208 trials, Supplemental Table 4). After a choice is made, participants receive feedback about the outcome of their choice. If a certain reward was chosen, they win the amount shown. If the gamble was chosen, the outcome of that gamble is shown. Participants are instructed, at the outset of the task, that one trial will be randomly selected at the end of the task and the outcome of that trial will contribute to their bonus payment of actual money. On one one-third of randomly selected trials (via computer random number generator), participants are shown a screen that asks, “How do you feel about the last outcome?” and are to enter their answer using a slider bar labeled from “very bad” to “very good”. The slider bar entry is encoded as a number between −4 to 0 to + 4 in whole units. Participants completed as many trials as they could within a 30-min window. On average, participants completed 208.65 ± 18.33 trials during each visit (Supplementary Table 4).

#### Statistical analysis and computational modeling of behavior

All statistical analyses were completed using RStudio^33^. Model parameters were determined using the RStan, rstanarm, and hBayesDM packages to perform hierarchical Bayesian modeling using task parameters and participant behavior^32–34^. The use of a hierarchical Bayesian model allowed us to produce both group-level and individual-level parameters, and allowed us to incorporate ICD and medication status as prior information about participant’s behavior in the model fitting. The difference in gambling rate was compared using the Wilcoxon Rank Sum Test. Individual participant behavior was fit using hierarchical Bayesian methods.

Two models were fit to participant behavior in our analyses. First, we fit a model (Eq. 1) following Rutledge et al.^16,17,32^, to determine how elements of each trial contributed to each participants’ rating of subjective ‘happiness’ with the outcome of their decisions:

1 $$Happiness\left(t\right)={w}_{0}+{w}_{1}\sum_{j=1}^{t}{\gamma }^{t-j}C{R}_{j}+{w}_{2}\sum_{j=1}^{t}{\gamma }^{t-j}E{V}_{j}+{w}_{3}\sum_{j=1}^{t}{\gamma }^{t-j}RP{E}_{j}$$

where on each trial $t$, the subjective happiness with the outcome is fit to a linear combination of the current trials’ values: $CR$ = Value of chosen Certain Rewards, $EV$ = Expected Value of Chosen Gambles, and $RPE$ = Reward Prediction Error. ${w}_{0}$ ${w}_{1}$, ${w}_{2}$ , ${w}_{3}$ are weights capturing the influence of each factor. $\gamma$ is a forgetting factor^16,17,32^ that increases the weight of recent events over events that occur further back in time. ${w}_{0}$ ${w}_{1}$, ${w}_{2}$ , ${w}_{3}$ , and $\gamma$ are fit to each participants’ subjective rating behavior. Subjective ratings were z-scored for each participant to account for individual variabilities in ratings. The model was fit to the ICD on, ICD off, non-ICD on, and non-ICD off data separately to generate group parameters for each condition. Model fits were evaluated by the r^2^ values of participants’ ratings and the model’s predicted ratings based on individual-level parameters (Fig. 2).

In the second model, values of participants’ subjective ratings in the previous trial and the expected values of the presented options in the current trial were used in Eq. 2 to determine the influence of feelings and objective parameters on participants’ decision to gamble or take the certain reward on each trial $t$:

2 $${\text{ln}}\left(\frac{P\left(Gamble\left(t\right)=1\right)}{P\left(Gamble\left(t\right)=0\right)}\right)={\beta }_{0}+ {\beta }_{1}EV\_G\left(t\right)+{\beta }_{2}EV\_CR \left(t\right)+ {\beta }_{3} SR\_H(t-1)$$

Here, we included the objective values: the expected value of the certain reward (“$EV\_CR$”) and the expected value of the gamble (“$EV\_G$”), and the reported subjective rating of happiness (“$SR\_H$”) with the outcome of the prior trial ($t-1$). Only trials that were preceded by a rating question were used to fit this model. ${\beta }_{0}$, ${\beta }_{1}$, ${\beta }_{2}$, and ${\beta }_{3}$ are weights capturing the influence of each factor. Additional details of the modeling approach are provided in the Supplemental Methods.

### Supplementary Information

Supplementary Information.

## Data Availability

The de-identified data and the analysis scripts used to produce the figures and tables in this publication are available on GitHub (https://github.com/KishidaLAB/Article-Subjective-Feelings-in-ICD).
