# Supplementary material for: Subjective feelings associated with expectations and rewards during risky decision-making in impulse control disorder
Source: Sci Rep. 2024 Mar 4;14:4627. doi: 10.1038/s41598-024-53076-2 (PMC10912783; doi:10.1038/s41598-024-53076-2)
Supplement: Supplementary file 1 — Supplementary Information. [file 41598_2024_53076_MOESM1_ESM.docx]

**Title:** Self-reported ratings about risky decisions detect dopamine-induced impulse control disorders

**Authors:** Brittany Liebenow^a,b^, B.A.; Angela Jiang^b^, B.S.; Emily K. DiMarco^a,b^, M.S.; Lester Paul Sands^a,b^; B.S., Mary Moya-Mendez^c^, M.S.; Adrian W. Laxton^d^, M.D.; Mustafa S. Siddiqui^d,e^, M.D.; Ihtsham ul Haq^f^, M.D.; Kenneth T. Kishida^a,b,d^, Ph.D.

**Affiliations:**

^a^ Neuroscience Graduate Program, Wake Forest School of Medicine, Winston-Salem, NC, United States of America

^b^ Department of Translational Neuroscience, Wake Forest School of Medicine, Winston-Salem, NC, United States of America

^c^ Duke School of Medicine, Durham, NC, United States of America

^d^ Department of Neurosurgery, Wake Forest School of Medicine, Medical Center Boulevard Winston-Salem, NC 27157

^e^ Department of Neurology, Wake Forest School of Medicine, Medical Center Boulevard Winston-Salem, NC 27157

^f^ Department of Neurology, University of Miami Miller School of Medicine, Miami, FL, United States of America

Corresponding Author:
Dr. Kenneth T. Kishida, Ph.D.
Department of Translational Neuroscience, Department of Neurosurgery
Wake Forest School of Medicine
Bowman Gray Center for Medical Education, 475 Vine Street
Winston-Salem, North Carolina, 27101, United States of America
Tel: 336-716-0419
E-mail: kkishida@wakehealth.edu

**SUPPLEMENTAL METHODS**

**Risky Decision-Making Task**

On any given trial there is a 50% chance that the gamble option is rationally the better option. For some trials this is obvious (e.g., 50% chance of winning $6 or $5 versus a certain outcome of $1); these trials provide control conditions to ensure participants are paying attention and performing the task as expected. On more equivalently valued trials (e.g., 50% chance of winning $6 or $2 versus a certain outcome of $4) the influence of baseline propensity to gamble and the influence of subjective feelings induced by recent trials can be observed.

**Participants**

18 patients with ICD and 12 without ICD took part in the study. In Supplemental Table 1, we report detailed information for each patient identified as ICD or non-ICD, the age, sex, order of on versus off medication testing visits, and standard of care prescribed dopaminergic medications are reported. Statistical tests show demographic and medication information between the non-ICD and ICD groups did not significantly differ.

**Hierarchical Bayesian Modeling of Subjective Feelings:**

An established computational model to estimate predictors of subjective feelings^1,2,3^ was fit to the data simultaneously for each individual and for each group using the following equation to model participants’ ratings:

$$Happiness\left( t \right)=w_{0}+w_{1}\sum_{j=1}^{t} \gamma^{t-j}CR_{j}+w_{2}\sum_{j=1}^{t} \gamma^{t-j}EV_{j}+w_{3}\sum_{j=1}^{t} \gamma^{t-j}RPE_{j}$$

Here, $t$ is the current trial number; $w_{0}$ is a constant term; $w_{1}$, $w_{2}$ , $w_{3}$ are weights capturing the influence of different event types: CR = Value of Chosen Certain Rewards, EV = Expected Value of Chosen Gambles, and RPE = Reward Prediction Error where the RPE is the reward outcome minus the expected value of the choice (CR if the certain reward option is picked, or EV if the gamble option is selected).^2,4^ 0 ≤ $\gamma$ ≤ 1 can be thought of as a ‘forgetting factor’ that preferentially weights recent event over events that occurred further back in time.^1,2,3^

We employed a hierarchical Bayesian approach to fit the group-level and individual-level models using the rstan^5,6^ package and a modified version of the Happiness Computational Model script (rdt_happiness) from hBayesDM^3^ in RStudio.^7^ Separate models were fit for the ICD on medication, the ICD off medication, the non-ICD on medication, and the non-ICD off medication data. We conducted Bayesian analysis on the resulting group means.

For each of the 4 conditions, we fit the happiness model using 8 sampling chains, each with 4,500 iterations that included 1,500 warm-up samples. The warm-up samples were excluded from the posterior distribution, leaving 24,000 samples for each parameter's posterior distribution. Traceplots for each parameter in each subject as well as the groups were visually inspected to ensure the chains had properly mixed and converged. We also examined R-hat, a convergence diagnostic for within-chain and between-chain variance. All R-hat values were less than 1.1 indicating the chains had converged. ^8^

For comparing and reporting the group posterior distributions, we used Kruschke’s recommendations as a guide^9,10^. Highest density intervals were calculated using the HDIofMCMC function from HBayesDM^3^ package, and Cohen’s *d* calculations were done using the effsize^11^ package in R. Frequentist group-level summary statistics (ICD versus non-ICD) were calculated using the parameters fit for each individual model.

**Gamble Choice Model:**

We used these subjective feelings ratings from the prior trial and objective trial values (i.e., the expected value of the gamble and value of the certain reward) from the corresponding trial to fit a gamble choice logistic model to determine what factors predict participants’ likelihood of choosing the gamble:

$$\ln\left( \frac{P\left( Gamble Choice\left( t \right)=1 \right)}{P\left( Gamble Choice\left( t \right)=0 \right)} \right)=\beta_{0}+ \beta_{1}Gamble EV\left( t \right)+\beta_{2}Certain Reward \left( t \right)+ \beta_{3}Imputed Subjective feeling(t-1)$$

The goal of this model was to determine whether subjective feelings elicited by the results of the prior trial could influence a participant’s decision to take a risk and gamble. Selecting a certain reward was coded as “0”, while selecting a gamble was coded as “1”; in this way, the calculated coefficients predict the likelihood of choosing the gamble option. This model was fit separately for each individual. Only trials preceded by a rating question were used to fit the model, as not all trials had associated ratings. Values for the ICD and non-ICD groups when on and off medication were computed by taking the average of each individual’s model coefficients for that particular group (ICD or non-ICD) and medication state (on or off). Data points identified as outliers through Grubb’s test were excluded from the calculation of group averages and subsequent t-tests. Outliers were identified based on a significance level of a p-value less than 0.05, with any data point exceeding this threshold being treated as an outlier. The ICD off and non-ICD on groups both had one individual whose coefficients for $\beta_{0}$, $\beta_{1}$, $\beta_{2}$, and $\beta_{3}$ were found to be outliers. In the ICD on group, an outlier was observed in the coefficient for $\beta_{3}$. In the non-ICD off group, the coefficients for $\beta_{1}$, $\beta_{2}$, and $\beta_{3}$ for a single individual were identified as outliers.

**SUPPLEMENTAL RESULTS**

**Task earnings are similar across ICD and non-ICD groups**

There were no significant differences across groups in number of trials in which participants chose to gamble (ANOVA, p = 0.98). There were no group differences in average reward value for both the certain reward trials (ANOVA, p = 0.92) and the gamble trials (ANOVA, p = 0.67). These results rule out alternative explanations for the group differences seen in the subjective feelings model.

Looking within the non-ICD cohort there were group differences across certain reward versus gamble and on versus off medication status (ANOVA, p = 7.7e-10) that were maintained in the ICD cohort (ANOVA, p = 5.1e-10). Within the non-ICD, certain rewards had greater average values (4.483, 95% CI 4.358 to 4.608) than gamble rewards (3.786, 95% CI 3.589 to 3.982) off medication (Tukey, p < 0.001); certain rewards (4.433, 95% CI 4.254 to 4.611) were also greater than gamble rewards (3.619, 95% CI 3.376 to 3.862) on medication (Tukey, p < 0.001). Likewise, these patterns of significant differences were found in the ICD cohort: certain rewards had greater average values (4.391, 95% CI 4.198 to 4.583) than gamble rewards (3.650, 95% CI 3.442 to 3.859) off medication (Tukey, p < 0.001); certain rewards (4.437, 95% CI 4.222 to 4.651) were also greater than gamble rewards (3.625, 95% CI 3.432 to 3.819) on medication (Tukey, p < 0.001).

**Supplementary References**

1. Rutledge, R. B., Skandali, N., Dayan, P. & Dolan, R. J. A computational and neural model of momentary subjective well-being. *Proceedings of the National Academy of Sciences* **111**, 12252-12257, doi:10.1073/pnas.1407535111 (2014).

2. Rutledge, R. B. *et al.* Association of neural and emotional impacts of reward prediction errors with major depression. *JAMA Psychiatry* **74**, 790-797, doi:10.1001/jamapsychiatry.2017.1713 (2017).

3. Ahn, W. Y., Haines, N. & Zhang, L. Revealing Neurocomputational Mechanisms of Reinforcement Learning and Decision-Making With the hBayesDM Package. *Comput. Psychiatry* **1**, 24-57, doi:10.1162/cpsy_a_00002 (2017).

4. Rutledge, R. B., Skandali, N., Dayan, P. & Dolan, R. J. Dopaminergic Modulation of Decision Making and Subjective Well-Being. *J. Neurosci.* **35**, 9811-9822, doi:10.1523/jneurosci.0702-15.2015 (2015).

5. Goodrich, B., Gabry, J. & Brilleman, S. rstanarm: Bayesian Applied Regression Modeling via Stan. *R package version* (2019).

6. Stan Development Team. Stan User’s Guide, Version 2.27. *Interaction Flow Modeling Language* (2020).

7. R Studio Team. R Studio. *R.S. ed. http://www.rstudio.com/.* (2020).

8. Gelman, A. & Rubin, D. B. Inference from Iterative Simulation Using Multiple Sequences. *Statistical Science* **7**, 457-472 (1992).

9. Kruschke, J. K. Bayesian estimation supersedes the t test. *J Exp Psychol Gen* **142**, 573-603, doi:10.1037/a0029146 (2013).

10. Kruschke, J. K. Bayesian Analysis Reporting Guidelines. *Nat Hum Behav* **5**, 1282-1291, doi:10.1038/s41562-021-01177-7 (2021).

11. Torchiano, M. effsize: Efficient Effect Size Computation. R package version 0.8.1, https://CRAN.R-project.org/package=effsize, doi:10.5281/zenodo.1480624 (2020)

**SUPPLEMENTAL TABLES**

**Supplemental Table 1: A) Demographic and Medication Information Per Patient.** For each patient identified as ICD or non-ICD, the age, sex, order of on versus off medication testing visits, and standard of care prescribed dopaminergic medications are reported.


 **Supplemental Table 1: B) Demographic and Medication Information for the Non-ICD and ICD groups.** An independent t-test was performed to test for differences in age between the ICD and non-ICD groups. Pearson’s chi-squared tests with Yates’ continuity correction were performed on the sex, the order of medication status visits, and medication categories. No tests were performed for Carbidopa/Levodopa as all participants were taking carbidopa/levodopa. ****

**Supplemental Table 2: Gambling rate in “Sure bet or Gamble” Task.** The mean percentage of gambles chosen (and 95% confidence intervals) for each group in each medication state. Wilcoxon Rank Sum Test results (p-value and W statistic) to determine significant differences across groups.

| **Gambling Percentage** |  |  |  |  | |
| --- | --- | --- | --- | --- | --- |
| **Group** | **Mean Gambling %** | **95% Confidence Interval** | | |  |
| **non-ICD Off** | 46.04 | 42.43 | to | 49.65 | |
| **non-ICD On** | 47.57 | 43.58 | to | 51.57 | |
| **ICD Off** | 47.00 | 42.32 | to | 51.69 | |
| **ICD On** | 44.97 | 40.76 | to | 49.19 | |
|  |  |  |  |  | |
| **Wilcoxon Rank Sum Test** |  |  |  |  | |
| **Comparison** | **P-Value** | **W Statistic** |  |  | |
| **ICD Off : non-ICD Off** | 0.9502 | 106 |  |  | |
| **ICD On : non-ICD On** | 0.3048 | 83 |  |  | |
| **non-ICD On : non-ICD Off** | 0.4023 | 87 |  |  | |
| **ICD On : ICD Off** | 0.7666 | 152 |  |  | |

**Supplemental Table 3: Gambling rate in trials where gambling was not optimal.** Gambling rate analysis restricted to trials where the value of the certain reward option was greater than the expected value of the gamble option. The mean percentage of gambles chosen (and 95% confidence intervals) for each group in each medication state. Wilcoxon Rank Sum Test results (p-value and W statistic) to determine significant differences across groups.

| **Gambling Percentage in trials where**  **(Value of the Certain Reward > Expected Value of the Gamble)** | | | | |
| --- | --- | --- | --- | --- |
| **Group** | **Mean Gambling %** | **95% Confidence Interval** | | |
| **non-ICD Off** | 12.38 | 8.38 | to | 16.39 |
| **non-ICD On** | 13.52 | 7.49 | to | 19.56 |
| **ICD Off** | 17 | 8.56 | to | 25.44 |
| **ICD On** | 15.22 | 9.15 | to | 21.28 |
|  |  |  |  |  |
| **Wilcoxon Rank Sum Test for trials where**  **(Value of the Certain Reward > Expected Value of the Gamble)** | | |  |  |
| **Comparison** | **P-Value** | **W Statistic** |  |  |
| **ICD Off : non-ICD Off** | 0.8822 | 112 |  |  |
| **ICD On : non-ICD On** | 0.8513 | 113 |  |  |
| **non-ICD On : non-ICD Off** | 0.931 | 74 |  |  |
| **ICD On : ICD Off** | 0.8993 | 157.5 |  |  |

**Supplemental Table 4: a)** Number of trials completed by each patient during each visit sorted by the medication status of the visit. **b)** A one-tailed paired t-test revealed patients completed significantly more when on versus off their dopaminergic medication.
